# Intracerebral Haemorrhage Segmentation in Non-Contrast CT

**DOI:** 10.1038/s41598-019-54491-6

**Published:** 2019-11-28

**Authors:** Ajay Patel, Floris H. B. M. Schreuder, Catharina J. M. Klijn, Mathias Prokop, Bram van Ginneken, Henk A. Marquering, Yvo B. W. E. M. Roos, M. Irem Baharoglu, Frederick J. A. Meijer, Rashindra Manniesing

**Affiliations:** 1 Department of Radiology and Nuclear Medicine, Radboud University Medical Center, 6525 GA Nijmegen, The Netherlands; 2 Department of Neurology, Radboud University Medical Center, 6525 GA Nijmegen, The Netherlands; 3 Biomedical Engineering & Physics Department, Amsterdam University Medical Center, University of Amsterdam, 1105 AZ Amsterdam, The Netherlands; 4 Department of Radiology and Nuclear Physics, Amsterdam University Medical Center, University of Amsterdam, 1105 AZ Amsterdam, The Netherlands; 5 Department of Neurology, Amsterdam University Medical Center, University of Amsterdam, 1105 AZ Amsterdam, The Netherlands

**Keywords:** Brain imaging, Computed tomography, Stroke, Computer science

## Abstract

A 3-dimensional (3D) convolutional neural network is presented for the segmentation and quantification of spontaneous intracerebral haemorrhage (ICH) in non-contrast computed tomography (NCCT). The method utilises a combination of contextual information on multiple scales for fast and fully automatic dense predictions. To handle a large class imbalance present in the data, a weight map is introduced during training. The method was evaluated on two datasets of 25 and 50 patients respectively. The reference standard consisted of manual annotations for each ICH in the dataset. Quantitative analysis showed a median Dice similarity coefficient of 0.91 [0.87–0.94] and 0.90 [0.85–0.92] for the two test datasets in comparison to the reference standards. Evaluation of a separate dataset of 5 patients for the assessment of the observer variability produced a mean Dice similarity coefficient of 0.95 ± 0.02 for the inter-observer variability and 0.97 ± 0.01 for the intra-observer variability. The average prediction time for an entire volume was 104 ± 15 seconds. The results demonstrate that the method is accurate and approaches the performance of expert manual annotation.

## Introduction

Non-traumatic intracerebral haemorrhage (ICH) is the most deadly stroke subtype^1^. Accurate segmentation and quantification of ICH has become increasingly important, as haemorrhage volume predicts patient outcome and haemorrhage features can aid in identifying etiology and guide secondary imaging and treatment decisions^2,3^.

**Figure 1:**
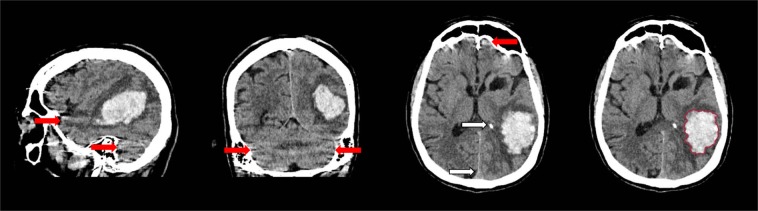
Sagittal, coronal and axial view of intracerebral haemorrhage with internal heterogeneity and irregular boundaries. Linear high density regions are caused by streaking artefacts as a result of beam hardening (red arrows). Intracranial calcifications and structures such as the falx cerebri share similar density values (white arrows). Haemorrhage boundary manually defined by a trained observer (right).

Computed Tomography (CT) imaging is the preferred modality for the initial assessment of patients presenting with symptoms of stroke in the majority of health care settings because it is readily available, can be performed fast and has high sensitivity for haemorrhage^4^. Generally, the appearance of haemorrhage on CT in the acute phase is distinct due to the hyperdensity of blood. However, segmentation of ICH can be challenging because of image noise, artefacts and cerebral parenchyma with similar appearance and density. Irregularity of the haematoma and different stages of clot formation may further contribute to obscure haemorrhage boundaries and internal heterogeneity. These difficulties are illustrated in Fig. 1.

The clinically widely adopted ABC/2 method considers perpendicular diameter measurements of the haemorrhage in both axial and coronal planes to estimate the total ICH volume^5^. Several studies have investigated the difference in haemorrhage volume estimation between ABC/2 methods and semi-automatic planimetric software^6–14^. These studies failed to demonstrate the difference in volume estimation in comparison to precise manual annotation, as they used semi-automatic methods requiring user interaction to serve as reference standard. Nevertheless, it has been demonstrated that the ABC/2 method is prone to observer variability and imprecise and shown to overestimate ICH volume by approximately 20%^6,7^. Large, irregular shaped haemorrhages are most prone to misestimation^7^. Despite the shortcomings of the ABC/2 method, it remains widely applied. Its continuous use may likely be attributed to its relative ease of use.

Automated evaluation of cranial non-contrast CT (NCCT) exams is a broad topic of interest that has resulted in methods for various applications. Recently, several large-scale studies have specifically focused on the detection of intracranial haemorrhage in an acute setting, with the aim of optimising clinical workflow and reducing time to diagnosis^15–18^. Related work on segmentation in cranial NCCT has mainly presented methods that operate in 2D and are not proposed for a specific subtype of intracranial haemorrhage. Although haemorrhage shape and appearance can strongly differ, even more so between subtypes. An overview of related work on cranial CT segmentation is shown in Table 1.

**Table 1:** Overview of related work on segmentation in cranial NCCT.

Author	Year	Method	*n*D	Pathology	Reference standard	Observer variability	Test set (N)		
							*Patients*	*Scans*	*Slices*
Bardera *et al*.^29^	2009	Semi-automated region growing and level set	—	ICH and edema	Slice-based manual†	Inter	18	18	—
Liao *et al*.^30^	2009	Multi-resolution binary level set	2D	ICH, SDH and EDH	Manual†	No	48	48	48
Prakash *et al*.^31^	2012	Modified distance regularised level set evolution	2D	ICH and IVH	Slice-based manual	No	—	50	—
Prakash *et al*.^32^	2012	Modified thresholding, clustering, and graph theory	2D	ICH and IVH	Slice-based manual	No	—	51	—
Bhadauria *et al*.^33^	2013	Fuzzy C-mean clustering and active contour	2D	ICH, SDH and EDH	Manual†	No	20	—	100
Boers *et al*.^34^	2014	Atlas registration and region growing	2D	SAH	Manual	Inter	30	30	—
Gillebert *et al*.^38^	2014	Template registration and statistical analysis	2D	Stroke lesions	Slice-based manual	No	24	24	—
Scherer *et al*.^39^	2016	Random forest classifier	—	ICH	Manual/Semi-automatic	Inter	30	30	—
Shahangian *et al*.^35^	2016	Modified distance regularised level set evolution	2D	ICH, IVH, SDH and EDH	Manual†	No	—	—	627
Gautam *et al*.^36^	2018	Fuzzy clustering and distance regularized level set evolution	2D	ICH	Manual†	No	—	—	60
Chang *et al*.^37^	2018	Convolutional neural network†	2D	ICH, SDH, EDH and SAH	Semi-automatic†	No	—	862 (82)	23,668 (—)
Muschelli *et al*.^40^	2017	Random forest classifier	3D†	ICH	Manual/Semi-automatic	No	102	102	—
This work	2019	Convolutional neural network	3D	ICH	Manual	Inter & Intra	30/—	30/50	—

Information that has not explicitly been specified in the cited publication is indicated by —. Information that has not clearly been explained in detail is indicated by †. The size of the test set is shown as the total number of 2D slices or 3D scans taken from a specified number of unique patients that was used for quantitative evaluation of the method. For example, Liao *et al*. included a single scan for each unique patient and from each scan a single slice was taken for evaluation. Numbers in parentheses indicate the number of cases containing pathology in a test set also containing healthy subjects.

In recent years, the use of convolutional neural networks (CNNs) in medical image analysis has shown a rapid increase^19^. For image segmentation, multiple CNN architectures have recently been presented, which show a general similarity and employ a combination of context information integration from multiple scales for simultaneous volumetric dense prediction^20–22^. In the field of medical image analysis, CNNs have already shown to rival or even surpass the performance of experienced medical professionals^23–25^.

We employed a state-of-the-art 3D CNN that combines multi-scale information with a 3D fully convolutional architecture for fast, automatic segmentation of ICH in cranial CT exams. The method was evaluated on two manually annotated datasets comprised of both supra- and infra-tentorial ICH.

## Results

### Observer variability

**Figure 2:**
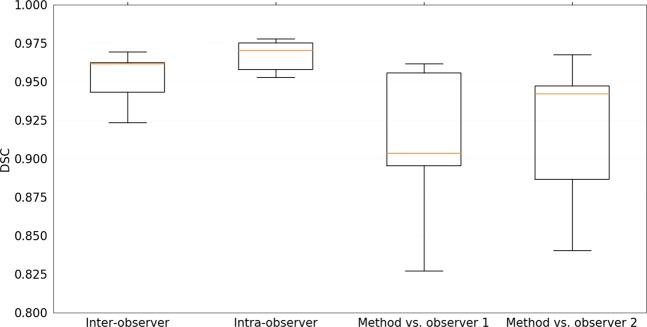
Box plots of Dice similarity coefficient (DSC) for inter-, intra-observer variability, method in comparison to each independent observer and final test dataset. Corresponding mean standard deviation values are listed in Table 2. The central lines show the median values, the box edges depict the 25th and 75th percentiles and the whiskers indicate the extremes at 1.5 interquartile range.

The quantitative evaluation of the inter-observer variability, intra-observer variability and performance of the method in comparison to each observer independently for a subset of five patients is shown in Tables 2 and 3. Results are reported for Dice similarity coefficient (DSC), Hausdorff Distance (HD), 95 percentile HD (95% HD), modified HD (MHD), contour mean distance (CMD) and absolute volume difference (AVD)^26–28^. The median and interquartile range are reported for non-normal distributions. A mean DSC of 0.95 ± 0.02 was recorded for the inter-observer variability. The performance of the method was at a comparable level to the observer variability, with mean DSC values of 0.91 ± 0.05 and 0.92 ± 0.05. No statistically significant difference (*p* < 0.05) in all metrics was found between the method and the inter-observer variability for the subset of patients. The results for DSC of all experiments are depicted in the box plots in Fig. 2.

**Table 2:** Quantitative evaluation of inter-, intra-observer variability, method in comparison to each independent observer and final test dataset reported as mean ± standard deviation or median and interquartile range for non-normal distributions.

	Inter- observer (n = 5)	Intra- observer (n = 5)	Method vs. observer 1 (n = 5)	Method vs. observer 2 (n = 5)
DSC	0.95 ± 0.02	0.97 ± 0.01	0.91 ± 0.05	0.92 ± 0.05
HD (mm)	6.14 [3.36–22.96]	6.59 [2.92–22.25]	8.03 ± 4.28	13.51 ± 13.29
95% HD (mm)	0.32 ± 0.30	0.00 [0.00–0.45]	1.69 ± 1.81	1.61 ± 1.79
MHD (mm)	0.07 ± 0.04	0.03 ± 0.01	0.26 ± 0.26	0.24 ± 0.23
CMD (mm)	0.28 ± 0.14	0.12 [0.12–0.18]	0.68 ± 0.52	0.57 ± 0.41
AVD (%)	5.49 ± 5.50	2.34 ± 1.92	11.64 ± 11.28	10.57 ± 9.76

Metrics reported are Dice similarity coefficient (DSC), Hausdorff distance (HD), 95 percentile HD (95% HD), modified HD (MHD), contour mean distance (CMD) and absolute volume difference (AVD). *p*-values were computed with a Wilcoxon signed-rank test between the inter-observer variability and method in comparison to the independent observers for each evaluation measure (columns 3 and 4). * indicates a significantly different result (*p* < 0.05).

**Table 3:** Quantitative evaluation of method in comparison to the reference standard for the Radboudumc test dataset reported as median and interquartile range.

DSC	0.91 [0.87–0.94]
HD (mm)	5.28 [3.80–12.11]
95% HD (mm)	0.79 [0.48–1.83]
MHD (mm)	0.17 [0.09–0.35]
CMD (mm)	0.49 [0.30–0.98]
AVD (%)	11.18 [7.30–17.89]

Metrics reported are Dice similarity coefficient (DSC), Hausdorff distance (HD), 95 percentile HD (95% HD), modified HD (MHD), contour mean distance (CMD) and absolute volume difference (AVD).

### Quantitative evaluation

#### Radboudumc

**Figure 3:**
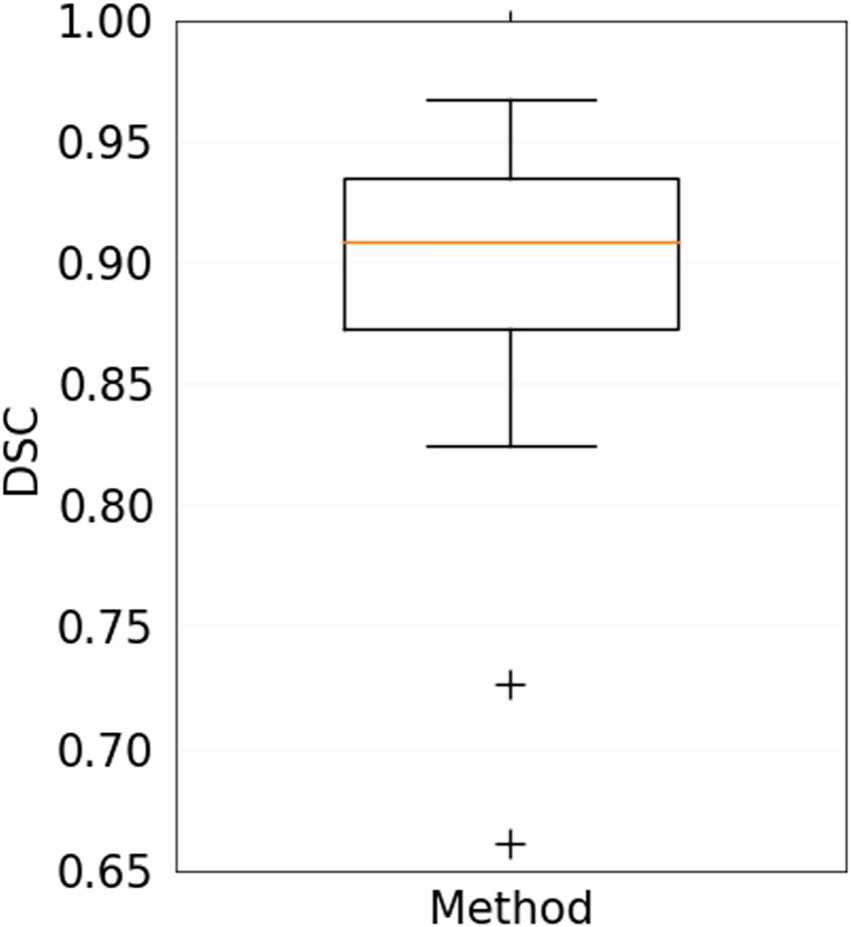
Box plots of Dice similarity coefficient (DSC) for method in comparison to the reference standard for the Radboudumc test dataset. Corresponding data are listed in Table 3. The central lines show the median values, the box edges depict the 25th and 75th percentiles and the whiskers indicate the extremes at 1.5 interquartile range excluding the two outliers, depicted as +.

Table 3 shows the results of the quantitative evaluation of the Radboudumc test dataset consisting of 25 patients. A median DSC of 0.91 [0.87–0.94] was reported. The results for DSC are depicted in a box plot in Fig. 3. The average prediction time for a single volume was 104 ± 15 seconds.

**Figure 4:**
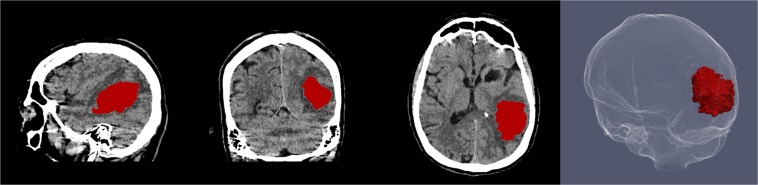
Sagittal, coronal and axial view of segmentation result of the proposed method for a patient with intracerebral haemorrhage with internal heterogeneity and irregular boundaries also depicted in Fig. 1. Three dimensional rendering of haemorrhage segmentation (right).

**Figure 5:**
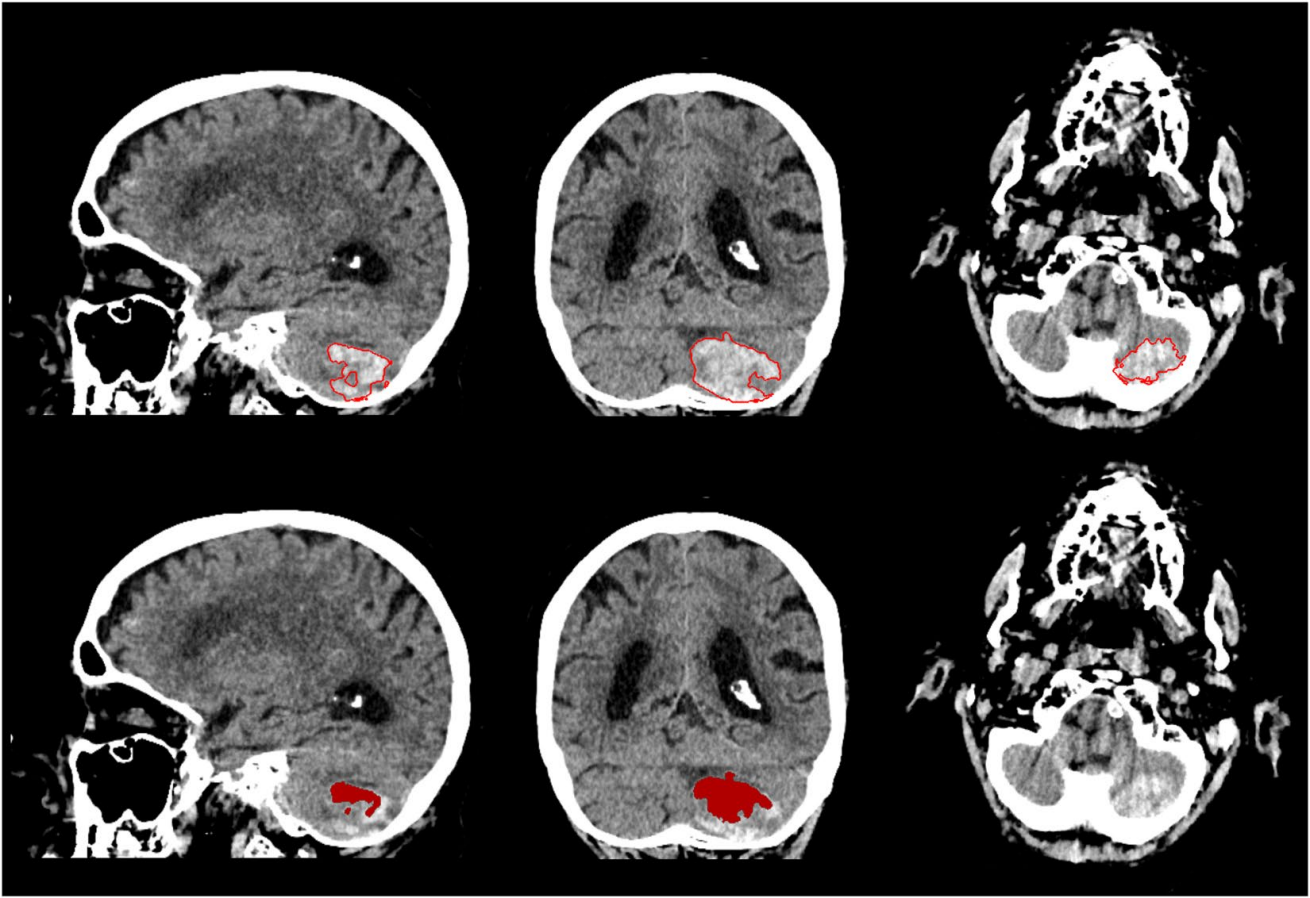
Sagittal, coronal and axial view of outlier of the test dataset depicted in Fig. 3. Patient with cerebellar haemorrhage close to the skull with high density streaking artefacts and internal heterogeneity. Manual annotation of ICH boundary (top) and segmentation result of the proposed method (bottom).

**Figure 6:**
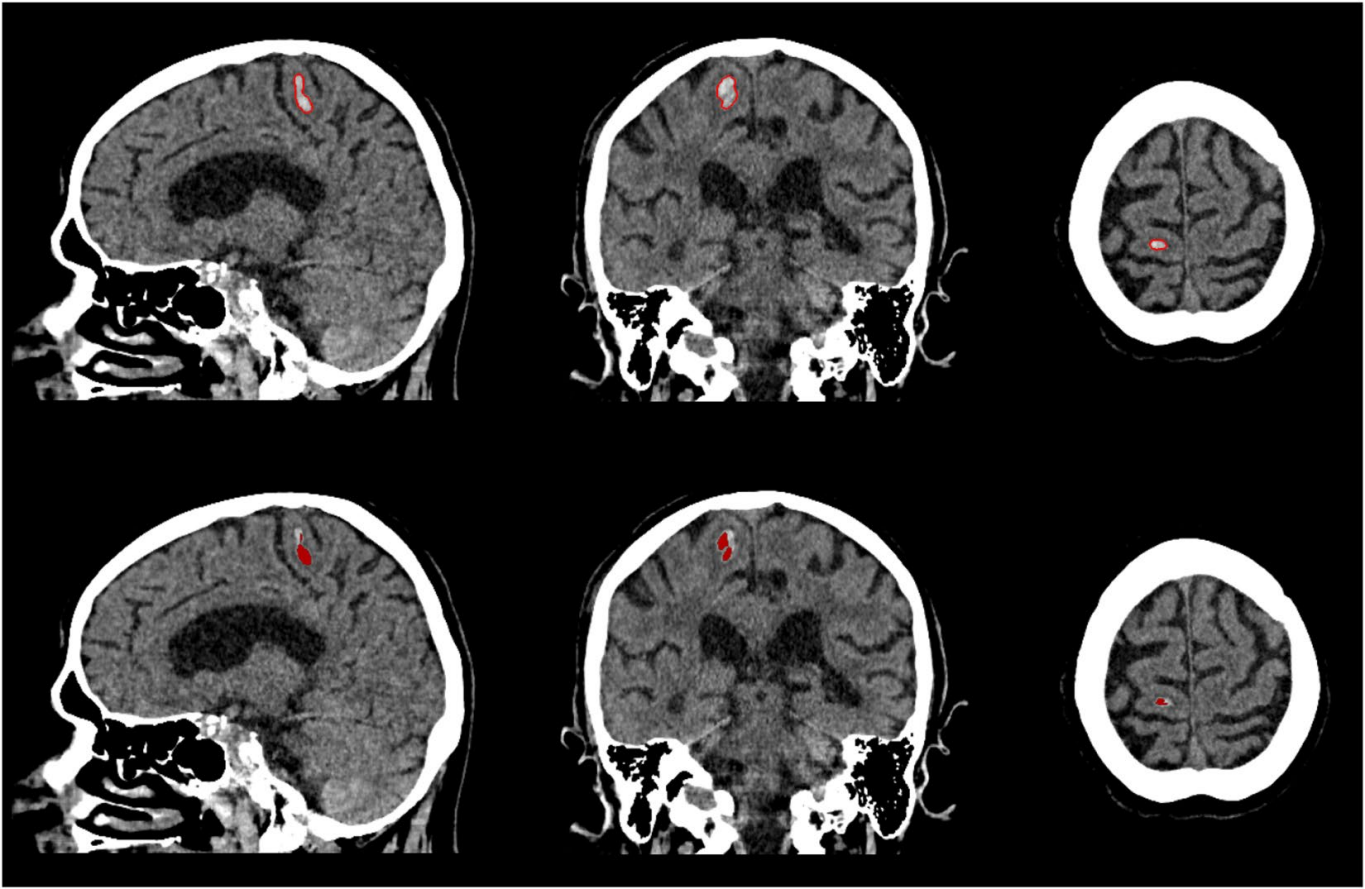
Sagittal, coronal and axial view of outlier of the test dataset depicted in Fig. 3. Patient with small haemorrhage in the right precentral gyrus. Manual annotation of ICH boundary (top) and segmentation result of the proposed method (bottom).

In general, the method was capable of segmentation of complex haemorrhages affected by image noise, heterogeneity due to varying stages of localised clotting and common imaging artefacts that may appear as haemorrhagic regions and cause false positive results, as shown in Figs. 1 and 4. However, two outliers that negatively affected the overall quantitative results could be identified. The first was a cerebellar haemorrhage located close to the occipital bone. This haemorrhage was affected by high intensity streaking artefacts causing heterogeneity with high intensity spots within the lower parts of the haemorrhage that resembled the surrounding skull. This artefact proved too severe for the method, resulting in a substantial undersegmentation in the final result. The second was a small intracerebral haemorrhage in the right precentral gyrus. A slight undersegmentation of the final result had a large impact on the quantitative measures because of the small volume of the haemorrhage. Both outlier results are depicted in Figs. 5 and 6.

#### PATCH

Table 4 shows the results of the quantitative evaluation of the separate PATCH test dataset consisting of 50 patients. A median DSC of 0.90 [0.85–0.92] was reported.

**Table 4:** Quantitative evaluation of method in comparison to the reference standard for the PATCH dataset reported as median and interquartile range.

DSC	0.90 [0.85–0.92]
HD (mm)	14.83 [5.38–53.10]
95% HD (mm)	0.43 [0.43–1.03]
MHD (mm)	0.12 [0.07–0.27]
CMD (mm)	0.24 [0.10–0.50]
AVD (%)	7.02 [3.07–15.69]

Metrics reported are Dice similarity coefficient (DSC), Hausdorff distance (HD), 95 percentile HD (95% HD), modified HD (MHD), contour mean distance (CMD) and absolute volume difference (AVD).

## Discussion

We have presented a method for fast and accurate automatic 3D segmentation of ICH in cranial NCCT. The method utilises a state-of-the-art deep learning approach that combines multi-scale context integration with a 3D fully convolutional architecture. This architecture allows for fast training and complex feature learning from volumetric data and negates the use of handcrafted features.

The method has proven to be accurate, with a median DSC of 0.91 [0.87–0.94] reported for the Radboudumc test dataset. The calculated median 95% HD of 0.79 [0.48–1.83] mm and median MHD of 0.17 [0.09–0.35] mm, further indicate that the segmentations produced by the method closely resemble the manually annotated reference standard with few erroneous over-segmented regions present elsewhere. Quantitative evaluation of the performance of the method using the PATCH dataset also showed a high DSC of 0.90 [0.85–0.92]. Although minor alterations to the network architecture and some retraining was necessary, the method has shown to be applicable to different datasets and produced good results. The variability of manual annotation between observers was investigated and showed a high degree of overlap, with a mean DSC of 0.95 ± 0.02. The method has shown to closely approach this level of human performance whilst reducing the time to perform the laborious task of segmentation. Statistical analysis showed no significant difference between the proposed method and the inter-observer variability. However, the small subset of five CT exams used is too small to draw definitive conclusions from. Nevertheless, the presented results provide an indication of the overall performance of the method.

We experienced difficulties with the method in two patients in the Radboudumc test dataset, as shown in Figs. 5 and 6. One of the patients had a total haemorrhage volume of less than 0.5 mL. In this case, a minor error in segmentation had a large impact on relative quantitative measures such as the DSC and AVD. Therefore, this negatively affected the overall quantitative results of the test dataset, as shown in Fig. 3. Another case was affected by image artefacts that largely impacted the appearance of the haemorrhage in terms of heterogeneity, leading to undersegmentation. However, the test dataset consisted of a variety of haemorrhages with differing shapes, internal heterogeneity and volumes, as shown in Table 5. Overall the method has proven to be robust to these varying factors.

**Table 5:** Overview of included patient data and haemorrhage characteristics divided into separate datasets for training and validation, and testing.

	Training and validation (n = 21)	Testing (n = 30)	
		Observer study (n = 5)	Evaluation (n = 25)
Male	10	2	15
Mean age (±SD)	65.2 ± 17.2	62.8 ± 23.4	66.8 ± 12.8
Median volume [IQ range]	13.7 [8.3–44.0]	20.6 [6.4–47.3]	4.1 [1.6–18.3]
Homo-/Heterogeneous	10/11	2/3	15/10
Regular/Irregular	8/13	1/4	14/11
Deep/Lobar	11/10	2/3	13/12

Related work concerning automatic methods has been limited to few studies in recent years. Eight methods shown in Table 1 employ region growing, level set evolution or clustering^29–36^. Region growing algorithms require selected intensity limits to constrain the final segmentation. Level set methods are sensitive to heterogeneity and the sharpness of lesion boundaries. These methods do not integrate surrounding context feature information for classification and are sensitive to image quality, haemorrhage appearance and artefacts. Six related works state that the reference standard for the quantitative evaluation was obtained by manual or semi-automatic annotation performed by an experienced observer, but neglect to provide details of how this was performed or which tools were used^29,30,33,35–37^. Furthermore, six studies only report the number of cases that were used for evaluation of the method and fail to clearly explain which data was used to develop the method^29,30,33,35,36,38^. Also, only three of the presented methods were evaluated in comparison to the variability of manual annotation^29,34,39^. One method employs voxel-wise classification using Random Forests that incorporates 3D neighbourhood information^40^. However, such an approach requires the selection and crafting of features that sufficiently describe the problem at hand. Therefore it is limited to the extent of the definition of those features. On the other hand, a CNN is capable of broad generalisation as a result of automated feature extraction and optimisation based on the data presented during training. Recent work using a CNN for intracranial haemorrhage detection and segmentation reported a DSC of 0.931 for ICH in subset of 23 patients^37^. However, the main focus of the work is the ROI-based backbone CNN architecture and no information is given about how the reference standard and final segmentations were obtained. Contrarily, our work clearly details all aspects of the methodology and evaluation and is compared to the inter- and intra-observer variability of manual annotation.

This work has a number of limitations. First, only patients with an isolated ICH were included in this study and those with extension of haemorrhage into the subarachnoid and intraventricular compartment were excluded. However, obtaining high-quality manually annotated reference standards is costly and laborious. Although the dataset for this study is limited, it has enabled the creation and validation of methodology for accurate ICH quantification in NCCT. Second, the method has shown to be capable of producing a volumetric segmentation in approximately 100 seconds. However, the method requires a pre-calculated segmentation of the cranial cavity to focus predictions within the diagnostically relevant region, which takes approximately six minutes. With a different training approach that also includes extracranial structures, the use of a cranial cavity segmentation may be circumvented in the future.

The focus of this work has been the accurate segmentation of ICH and the quantification of total haemorrhage volume. This is a fundamental step in haematoma characterisation. The segmentation of ICH may further aid in the identification of patients at high risk of ICH expansion, an independent predictor of early neurological deterioration and functional outcome. Several promising NCCT biomarkers have been investigated that could be used for patient stratification in clinical practice and trials, including margin irregularity, internal density heterogeneity and intrahaematoma fluid levels^41^. Therefore, future work may consist of extending current methodology to incorporate such features in the automated analysis. Furthermore, our method may form the basis of a method for automated detection of blood in cranial NCCT, which may aid in clinical practice to easily detect small amounts of blood that may otherwise be overlooked.

In conclusion, we presented an accurate and fast method for the automatic segmentation and quantification of ICH in NCCT using a 3D convolutional neural network that has been well validated in comparison to a manually annotated reference standard. The performance of the method approaches the variability of expert manual annotation, making it suitable for use in clinical studies requiring accurate volumetric analysis.

## Methods

### Network architecture

**Figure 7:**
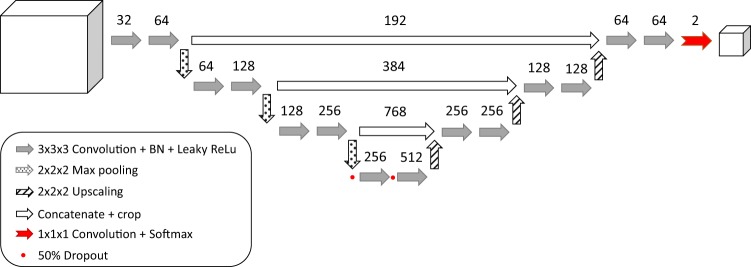
Schematic overview of CNN architecture. Arrows represent layers of filters. Numbers define the number of feature maps produced by the corresponding filter layer. The final softmax layer produces a probability map of the prediction for a given input at voxel level.

A schematic overview of the CNN architecture is shown in Fig. 7.

The proposed network architecture consists of two distinct pathways integrating contextual information on four different scales^21^. The first pathway is comprised of repeated units of convolutional filters and non-linearities followed by a regional intensity pooling operation to decrease the dimensionality of the feature maps and increase complexity in the lower scales of the network. A unit in the first pathway is made up of two layers of 3 × 3 × 3 convolutional filters, followed by a 2 × 2 × 2 pooling operation that takes the maximum value in that region and decreases the input feature map by a factor of eight. The number of filters is doubled between the first and second layer. This unit is repeated three times to create four separate scales within the architecture.

The purpose of the second pathway is twofold; it is an integrative path that incorporates feature information from all scales and it adds complexity and further features to the model through different units of convolutional filters that process the combined multi-scale information. The units of the second pathway are comprised of a 2 × 2 × 2 upscaling operation that increases the dimensionality of the input feature map by a factor of eight by regional repetition of the underlying values. This information is concatenated with the feature map on the same scale from the first pathway to combine both local and global feature information. The combined information from both pathways passes through two layers of 3 × 3 × 3 convolutional filters. The final combination of multi-scale feature information is transformed into an output probability map by a softmax function.

Differing from the original method, we implement leaky rectified linear units (ReLu) activation functions to prevent neuron saturation and sparse activations within the network^21,42^.

### Pre-processing

All images were normalised in the same manner. Intensity values below and above the relevant range were set to the scaled minimum and maximum values respectively. Values in the range [−200, 200] HU were normalised to the range [0, 1] to serve as input for the CNN. To aid in training the network and to prevent false positive predictions during the test phase, the diagnostically relevant cranial cavity was isolated. The cranial cavity is defined as all soft tissues and cerebrospinal fluid, including the meninges, cerebrum and ventricles, cerebellum and brain stem. This was segmented for each CT study using multi-atlas registration and levelset refinement^43^. Errors in the cranial cavity segmentation were manually corrected in nine cases.

### Training

The cranial cavity segmentation was combined with a manual annotation of each haemorrhage as described in Section *Reference standard*, to produce a full annotation mask for each CT image in the training set. Random coordinates were equally sampled from the annotated mask for haemorrhage (foreground) and cranial cavity (background) and used as centre point to extract volumetric patches of 124 × 124 × 124 voxels in the CT images and patches of 36 × 36 × 36 voxels in the annotated binary masks. The difference in dimensions between the input and output patches is a result of the accumulation of operations in the CNN architecture and is equal to the receptive field of a single voxel. This combination of tiles was used as input for the CNN during the training process.

The number of voxels that represented the background far exceeded the number of voxels representing haemorrhage within each image. Therefore, there was a class imbalance within the training data. If not taken into account, this may result in the model overfitting on the most prevalent class and becoming insensitive to the class of interest. To combat this problem, a weight map was used to balance the influence of both classes present in the data. For each training patch, the number of foreground voxels was determined and an equal number of background voxels was randomly selected within the patch. In this manner, a weight map was created for each patch that determined which voxels contributed to the calculation of the categorical cross entropy loss function. If the number of foreground voxels exceeded half of the training patch or the patch was solely comprised of background, all weights were set to one.

Data augmentation was used to increase the diversity of samples generated for training and thereby introduce a locational invariance to the network. During sampling, an augmentation probability of 50% was set. Augmented patches were either mirrored or rotated in the axial plane with a randomly selected angle in the range of [−15, 15] degrees.

Stochastic gradient descent was used to minimise the loss with a learning rate of 10^−3^ and Nesterov momentum of 0.9. Dropout^44^ of 50% was used in the lowest scale for regularization, as shown in Fig. 7. The network was trained on a total of 50000 training patches on an NVIDIA TitanX GPU. All network hyper-parameters were optimised during pilot experiments performed on the training and validation dataset. The method was developed using the Theano and Lasagne libraries^45,46^.

### Post-processing

For each case in the test dataset a segmentation of the haemorrhage was obtained by processing consecutive input tiles extracted from the image using the GPU described in Section *Training*. The final segmentation for the ICH was acquired by thresholding the output probability map at 0.5 and removing all clusters smaller than 0.1 mL in volume, as ICH comprises larger volumes. This threshold was determined by pilot experiments performed on the separate validation dataset.

## Patient data

### Radboudumc

This study was approved by the committee on ethics and research involving human subjects of the Radboud University Medical Center, Nijmegen, the Netherlands, and the requirement for informed consent was waived. All methods were performed in accordance with the relevant guidelines and regulations associated with the use of this data. Anonymised data was obtained by retrospectively searching our clinical-research image database for all adult patients with a supra- or infratentorial ICH that received a NCCT of the head at the Radboud University Medical Center, Nijmegen, the Netherlands between January 1^st^ 2012 and December 31^st^ 2016.

All images were visually inspected for quality. Exclusion criteria were the presence of blood outside of the brain parenchyma, ICH resulting from trauma, macrovascular lesions or malignancy and images with severe artefacts as a result of motion or presence of foreign objects. In total 51 patients were included, of which 21 were used for training and validation of the CNN. Of the remaining 30 scans, 25 were used to test the method and 5 were used to assess the observer variability. Haemorrhage characteristics were visually assessed by an experienced observer. ICH involving the deep nuclei or periventricular white matter was defined as deep ICH, haemorrhage involving the cerebral cortex was defined as lobar ICH. ICH was deemed heterogeneous when multiple clear patches of hypoattenuation were visible within the hyperattenuating haemorrhage. ICH with multiple protuberances and lacking a smooth lesion edge were deemed irregular. An overview of all study data, patients’ age and gender distributions, and haemorrhage characteristics is shown in Table 5.

### PATCH

An additional dataset consisting of 120 scans of Dutch patients and manual reference standard that were included in the Platelet transfusion versus standard care after acute stroke due to spontaneous cerebral haemorrhage associated with antiplatelet therapy (PATCH) trial in the period February 2009–October 2015 was obtained from the Amsterdam Medical Center, Amsterdam, the Netherlands^47^. Twenty cases with extension of haemorrhage into the intraventricular space or other compartments were excluded. The remaining cases in the dataset were divided into training, validation and test sets containing 40, 10 and 50 scans respectively.

### Acquisition protocol

All Radboudumc images were acquired using a 320-row Canon Aquilion ONE CT scanner manufactured by Canon Medical Systems Corporation, TMSC, Otawara, Japan. The CT protocol consisted of one head scan at 120 kV. CT image reconstruction was done with a FC25 or FC26 reconstruction kernel. The image sizes were 512 × 512 voxels and the number of slices was in the range [281–534] with voxel sizes of 0.43 × 0.43 × 0.5 mm.

The PATCH dataset primarily consisted of images acquired using a Siemens Sensation 64 at 120 kV. CT image reconstruction was performed with a H31s reconstruction kernel. The image sizes were 512 × 512 voxels and the number of slices was in the range [30–40] with voxel sizes of 0.53 × 0.53 × 5 mm.

### Reference standard

Manual annotations for the Radboudumc dataset were obtained to serve as a reference standard to train and validate the method. For each CT exam, the complete haemorrhage volume was annotated using a volumetric annotation tool VCAST, that utilises regional intensity clustering on multiple scales to expedite the laborious annotation process^48^. The local clustering allows for fast annotation of large volumes of similar intensity within the image. Haemorrhage boundaries were annotated on a voxel level to preclude bias or error introduced by the clustering algorithm of the annotation tool.

All manual annotations were performed by trained observers with a window width and level setting of 80/40 HU, supervised by a neuroradiologist (FJAM) with over ten years of experience.

### Evaluation metrics

All segmentations were evaluated by comparison to the manually annotated reference standard using the following metrics: Dice similarity coefficient (DSC), Hausdorff Distance (HD), 95 percentile HD (95% HD), modified HD (MHD), contour mean distance (CMD) and absolute volume difference (AVD). The mean and standard deviation for each measure was taken over all patients.

## Experiments

### Observer variability

A subset of the test data consisting of five CT exams was used to assess the variability of manual annotation between two trained observers. The five CT exams were annotated by both observers. Furthermore, the second observer annotated the same subset on a second occasion, two weeks apart, to estimate the intra-observer variability. The metrics described in Section *Evaluation metrics* were reported for the inter-observer variability, intra-observer variability and the performance of the method in comparison to each observer independently. Paired statistical tests were performed to test if the performance of the proposed method for the aforementioned evaluation metrics was significantly different (*p* < 0.05) from the inter-observer variability. A paired sample *t*-test was performed if the data was normally distributed according to the Shapiro-Wilk test. If not normally distributed, a Wilcoxon signed-rank test was performed.

### Quantitative evaluation

#### Radboudumc

For each of the 25 patients in the Radboudumc test dataset the final segmentation was compared to the manual reference standard. For each segmentation the metrics described in Section *Evaluation metrics* were reported.

#### PATCH

In order to perform quantitative evaluation on the additional PATCH dataset, some minor modifications were made to the original training scheme. The original training and validation data shown in Table 5 was resampled to an axial slice thickness of 5 mm. The network architecture shown in Fig. 7 was modified by removal of the max pooling and upsampling operations in the z-direction. The modified architecture was subsequently trained according to Section *Training* using the resampled training data and an input patch size of 124 × 124 × 40 voxels. Once an optimal performance on the resampled validation data was reached, training was suspended.

All scans and corresponding reference standards in the additional PATCH dataset were resampled to 0.43 × 0.43 × 5 mm voxel size. The training of the modified architecture was continued using the PATCH training data subset until an optimal performance was reached on the validation subset.

For each of the 50 scans in the PATCH test subset the final segmentation was compared to the manual reference standard. For each segmentation the metrics described in Section *Evaluation metrics* were reported.
